# FlyPNS, a database of the *Drosophila *embryonic and larval peripheral nervous system

**DOI:** 10.1186/1471-213X-5-4

**Published:** 2005-02-17

**Authors:** Virginie Orgogozo, Wesley B Grueber

**Affiliations:** 1 Department of Ecology and Evolutionary Biology, Princeton University, Princeton, NJ 08544, USA; 2 HHMI, Genetics, Development, and Behavioral Sciences Building GD481, 1550 4th Street, University of California, San Francisco, CA 94143-0725, USA

## Abstract

**Background:**

The embryonic and larval peripheral nervous system of *Drosophila melanogaster *is extensively studied as a very powerful model of developmental biology. One main advantage of this system is the ability to study the origin and development of individual sensory cells. However, there remain several discrepancies regarding the organization of sensory organs in each abdominal segment A1-A7.

**Description:**

We have constructed a web site called FlyPNS (for Fly Peripheral Nervous System) that consolidates a wide range of published and unpublished information about the embryonic and larval sensory organs. It communicates (1) a PNS pattern that solves the discrepancies that have been found in the recent literature, (2) the correspondence between the different nomenclatures that have been used so far, (3) a comprehensive description of each sensory organ, and (4) a list of both published and unpublished markers to reliably identify each PNS cell.

**Conclusions:**

The FlyPNS database integrates disparate data and nomenclature and thus helps understanding the conflicting observations that have been published recently. Furthermore, it is designed to provide assistance in the identification and study of individual sensory cells. We think it will be a useful resource for any researcher with interest in *Drosophila *sensory organs.

## Background

The *Drosophila *abdominal larval PNS is composed of a constant number of neurons and associated cells, whose characteristics and positions are reproducible between individuals [1-5]. Its stereotyped pattern has made it an ideal system to study many aspects of developmental biology such as cell determination, asymmetric cell divisions, cell lineages, development and remodeling of axons and dendrites, cell migration, and cell death.

Despite the extraordinary utility of the PNS for understanding these diverse problems, a prominent current limitation is the lack of agreement in the literature of the exact number, position and nomenclature of sensory neurons. For example, many different names have been used to describe abdominal neurons, reflecting the number of cells within a cluster, their position [6,7], their dendritic arborization [8,19] or their cell lineage origin [9]; and the correspondence between names of cells has remained vague. Furthermore, there has been no consensus on the exact number of neurons located in the dorsal cluster, which is one of the most extensively studied regions of the PNS. First studies indicated that 10 [4], 11 [2], or 12 [4,6] neurons are present. Following these studies, this cluster was usually thought to contain 12 neurons. A single compilation of the different nomenclatures that accounts for all peripheral neurons would not only benefit those within the field, but should also make it easier for researchers outside of the immediate field to comprehend the literature.

## Construction and content

We have constructed a website, named FlyPNS, that consolidates a wide range of published and unpublished information regarding the embryonic and larval sensory organs and their associated glial cells. Motoneurons and other glial cells have not been included. The FlyPNS web site is arranged in 6 sections: General references, Nomenclature, Abdominal PNS organization, Sensory organ description, Antibodies and enhancer-traps, Gal4 lines, and a Search option.

The **General references **section provides a list of papers on various aspects of sensory organ morphology and development. Subcategories include general descriptions, descriptions of dendritic arborizations, axonal pathways, developmental changes, and functions of multidendritic neurons. Links are provided to PubMed entries for access to abstracts or full-texts of papers.

**Figure 1:**
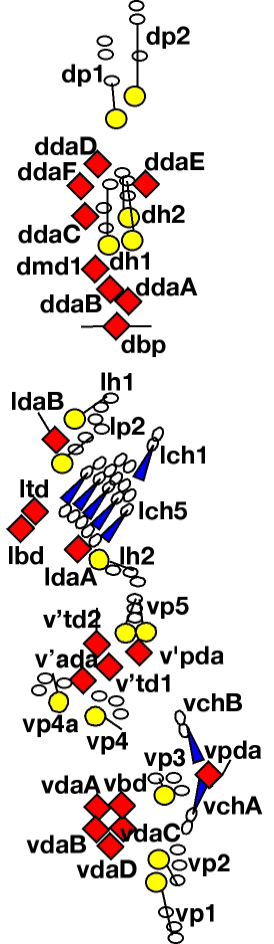
**Diagram of the sensory cells of an embryonic or larval abdominal hemisegment (A1-A7)**. Three types of sensory organs are found: (1) external sensory organs, composed of three accessory cells (oval shape) and one or several neurons (circular shape), (2) chordotonal organs, composed of accessory cells (oval shape) and neurons (elongated triangular shape), (3) multidendritic neurons (diamond shape). The most conspicuous sensory dendrites are represented as a straight line. Note that the dmd1/dda1 neuron presents a unique dendritic arborization different from the other da neurons. Anterior is left and dorsal is up.

In the **Abdominal PNS organization **section, we have represented the PNS pattern that we have observed (Fig. 1), with the notable presence of 13 neurons in the dorsal cluster (as also mentioned recently in [10,11]). In previous representations that indicated a smaller number of cells in the cluster, either the anterior-located Cut-negative neuron that we have named dmd1 (Fig. 1) or one of the Cut-positive multidendritic neurons may have been missed. Since we found that the precise position of sensory cells may vary with segment, embryo and developmental stage (unpublished data), the most reliable criteria for identification of *Drosophila *embryonic and larval sensory organs are marker expression and cell morphology, and these should be used whenever possible. Our study of the cell markers Cut, Collier, E7-2-36, E7-3-49, Elav, Engrailed and Nubbin/Pdm1 [11-13] and unpublished data), as well as neuron morphology [8,13], revealed that distinctive features can be attributed to each neuron, thereby allowing the unambiguous identification of every PNS neuron. The sections entitled Sensory organ description, Antibodies and enhancer-traps, and Gal4 lines relate these observations. It should also be noted that the precise geometry of the PNS pattern presented in the web site might vary somewhat depending on the stage examined and the techniques used to dissect and mount the preparation.

The section on **Nomenclature**, presented in table form, lists all nomenclatures that have been used in the literature, drawing explicit connections between the various names wherever possible (Table 1). This table is aimed to resolve inconsistencies in the literature, especially when particular cells have been omitted or subtle denominations interchanged. Many different names have been used for both the external sensory organs and the multidendritic neurons. The multidendritic neurons have been classified and named based on their gross morphology and the substrate upon which they extend their dendrites [14]. They were therefore called tracheal dendrite (md-td), bipolar dendrite (md-bd) and dendritic arborization (md-da) neurons. In recent papers, the "md" has often been dropped and they have been referred to as td, bd, or da neurons. Whereas the bd and td neurons usually reside singly and their identification is usually not problematic, the da neurons are found grouped as clusters of cells and it has been more difficult to assign individual identities. Early papers named these clusters according to the number of da neurons they contain. The dorsal cluster was therefore called dmd5 (for dorsal multidendritic cluster of 5) [15] or dmd6 [6]. Merritt and Whitington were the first to name nearly all da neurons as individuals [7] by providing each with an alphabetic designation reflecting their ventral-to-dorsal position. However, their dorsal cluster contained only 5 da neurons, and since we now know that this cluster typically contains an additional, sixth da neuron, it has not been possible to assign their specific names to the cluster. When Grueber et al. characterized the morphology of all da neurons [8], and Sweeney et al. the morphology of the dorsal cluster neurons [19], each group chose to assign names in the same vein as Merritt and Whitington. However, the typical relative positions of some neurons were not resolved because specific markers were lacking. Cut antibodies were subsequently used to discriminate the different neurons, and their typical relative positions were established [13].

**Table 1:** Correspondence between the nomenclatures previously used to name sensory organs or neurons.

	**[5]**	**[16]**	**[3] (names es organs)**	**[4] (names es and ch neurons)**	**[6] (names es, ch and md neurons)**	**[7] (names es, ch and md neurons)**	**[1] (names sensory organs)**	**[8] (names md da neurons)**	**other (Only the first paper using this other nomenclature is indicated)**
**external sensory (es) organs**	-	-	p1	vesA	vesA	vesA	vp1	-	vc1 [17]
	
	-	-	p2	vesB	vesB	vesB	vp2	-	vc2 [17]
	
	-	-	p3	vesC	vesC	vesC	vp3	-	vc3 [17]
	
	-	-	p4	v'esA	v'esA	v'esA	vp4	-	vc4 [17]
	
	-	-	p5	v'esB	v'esB	v'esB	vp4a	-	vc4a [17]
	
	-	-	p6	v'es2	v'es2	v'es2	vp5	-	vc5 [17]
	
	b	sensory hair C	h1	lesA	lesA	lesA	lh2	-	-
	
	-	-	p7	lesB	lesB	lesB	lp2, lc1	-	-
	
	H	sensory hair C	h2	lesC	lesC	lesC	lh1	-	-
	
	b+st	sensory hair F	h4	desB	des2	desA2	dh2	-	
	
			h3	desA	desB	desB	dh1	-	-
	
	s	-	p8	desC	desC	desC	dp1, dc1	-	-
	
	b	-	p9	desD	desD	desD	dp2, dc2	-	-

**chordotonal (ch) organs**	-	-	-	vchA	vchA	vchA	vch1	-	-
	
	-	-	-	vchB	vchB	vchB	vch2	-	-
	
	-	-	-	lch5	lch5	lch5	lch5	-	-
	
	-	-	-	v'ch1	v'ch1	v'ch1	lch1	-	-

**multidendritic (md) neurons**	-	-	-	-		vbd	vbd	-	vmd3 [9]
			
	-	-	-	-		vdaC		vdaC	vmd2 [9]
					
	-	-	-	-		vdaA		vdaD	vmd1 [9]
					
	-	-	-	-	vmd5	vdaD	vdaA-D	vdaA	vmd4 [9]
					
	-	-	-	-		vdaB		vdaB	vmd1a [9]
	
	-	-	-	-	vpda	vpda	vdap	vpda	-
	
	-	-	-	-	v'ada	v'ada	vdaa	v'ada	vmd4a [9]
	
	-	-	-	-				-	v'td1 [this paper]
		
	-	-	-	-	v'td2	v'td2	vtd1/2	-	v'td2 [this paper]
	
	-	-	-	-	v'pda	v'pda	v'dap	v'pda	-
	
	-	-	-	-	ldaA	ldaA	lda	ldaA	-
	
	-	-	-	-	lbd	-	isbp	-	ldb, lbd [18]
	
	-	-	-	-	ltd	ltd	istd	-	-
	
	-	-	-	-	ldaB	ldaB	ltd	ldaB	-
	
	-	-	-	-	dbd	dbd	dbp	-	-
	
	-	-	-	-		ddaA		ddaA	the ddaA-B-C-D-E cluster has also been called
						
	-	-	-	-		ddaB or ddaC?	ddaA-E	ddaB	dmd5 [15]
						
	-	-	-	-	dmd6	ddaB?		ddaC	
						
	-	-	-	-		-		ddaD	ddaF [19]
						
	-	-	-	-		ddaD?		ddaE	
						
	-	-	-	-		ddaD?		ddaF	ddaD [19]
	
	-	-	-	-	-	ddaE	-	-	dda1 [18]
									dmd1 [13]

Each nomenclature presented in Table 1 has its own advantages, and no single nomenclature is yet uniformly agreed upon by all researchers. The nomenclature presented on some FlyPNS pages, such as the "Sensory organ description" page, simply reflects the names that we use in our own work and that have also been found in several recent publications. We hope and think that with time researchers will agree on a common constructive nomenclature that also allows wide comprehension of the wealth of data already available on PNS development.

**Figure 2:**
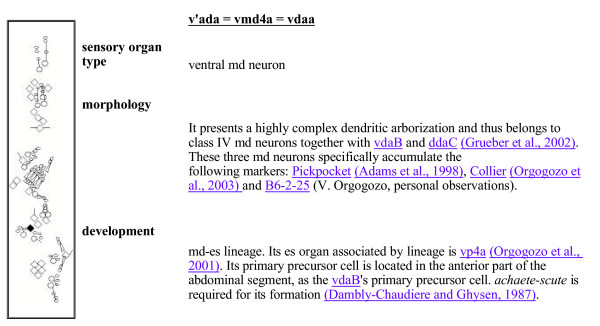
**Description of the sensory organ v'ada**. Links to other sensory organ descriptions, antibodies, enhancer-trap markers and references are shown. Clicking on a cell depicted in the diagram links to its sensory organ description.

The **Sensory organ description **section gives an extensive description for each individual sensory organ: position, name(s), markers, morphology, development and related references. A few unpublished observations relating to maker expression and morphology are also mentioned. A typical sensory organ description is shown in Fig. 2. Links to other sensory organ descriptions, antibodies, enhancer-trap markers and references are available. Clicking on the various cells depicted in the adjacent diagram also allows navigating between sensory organ descriptions. The extensive cross-linkage of the various pages of the web site is designed to facilitate the comprehension of the available data.

The **Antibodies, enhancer-traps and Gal4 lines **pages display a collection of published and unpublished data on expression patterns of antibodies, enhancer-traps and Gal4 lines, with links to the gene/insertion information contained in Flybase when available.

Finally, the **Search** option provides a convenient link to the information about any of the organs/markers.

## Utility, discussion and conclusions

Much of the discordance in the literature regarding the larval and embryonic PNS pattern seems to be rooted in variation in the precise position of sensory cells with segment, embryo, and time, and a lack of specific markers for some organs. These factors might explain why, for example, some dorsal cluster neurons were often missed in previous publications. We have attempted to resolve remaining ambiguities by (1) identifying the total number of sense organs and neurons in each abdominal segment A1-A7, (2) resolving different nomenclatures used for each organ, and (3) providing a collection of molecular markers and descriptions that provide an unambiguous guide for cell identification. This data compilation will hopefully also increase the accessibility of the fly PNS literature to non-*Drosophila *investigators.

## Availability

The FlyPNS web site is available from the link under Miscellaneous in the FlyBase Allied Data section . Comments and additions are welcomed by e-mail to VO.

## List of abbreviations

PNS: peripheral nervous system

da neuron: neuron displaying a dendritic arborization

## Authors' contributions

The PNS pattern was established based on the observations of WBG and VO. VO created the FlyPNS web site and WBG made significant improvements and corrections to the web site. Both VO and WBG wrote the manuscript.
